# Velocity prediction of nanofluid in a heated porous pipe: DEFIS learning of CFD results

**DOI:** 10.1038/s41598-020-79913-8

**Published:** 2021-01-13

**Authors:** Meisam Babanezhad, Iman Behroyan, Azam Marjani, Saeed Shirazian

**Affiliations:** 1 Institute of Research and Development, Duy Tan University, Da Nang, 550000 Vietnam; 2 Faculty of Electrical–Electronic Engineering, Duy Tan University, Da Nang, 550000 Vietnam; 3 Department of Artificial Intelligence, Shunderman Industrial Strategy Co., Tehran, Iran; 4 Faculty of Mechanical and Energy Engineering, Shahid Beheshti University, Tehran, Iran; 5 Department of Computational Fluid Dynamics, Shunderman Industrial Strategy Co., Tehran, Iran; 6 Department for Management of Science and Technology Development, Ton Duc Thang University, Ho Chi Minh City, Vietnam; 7 Faculty of Applied Sciences, Ton Duc Thang University, Ho Chi Minh City, Vietnam; 8 Laboratory of Computational Modeling of Drugs, South Ural State University, 76 Lenin prospekt, 454080 Chelyabinsk, Russia

**Keywords:** Astronomy and planetary science, Energy science and technology, Nanoscience and technology

## Abstract

Utilizing artificial intelligence algorithm of adaptive network-based fuzzy inference system (ANFIS) in combination with the computational lfuid dynamics (CFD) has recently revealed great potential as an auxiliary method for simulating challenging fluid mechnics problems. This research area is at the beginning, and needs sophisticated algorithms to be developed. No studies are available to consider the efficiency of the other trainers like differential evolution (DE) integrating with the FIS for capturing the pattern of the simulation results generated by CFD technique. Besides, the adjustment of the tuning parameters of the artificial intelligence (AI) algorithm for finding the highest level of intelligence is unavailable. The performance of AI algorithms in the meshing process has not been considered yet. Therfore, herein the Al_2_O_3_/water nanofluid flow in a porous pipe is simulated by a sophisticated hybrid approach combining mechnsitic model (CFD) and AI. The finite volume method (FVM) is employed as the CFD approach. Also, the differential evolution-based fuzzy inference system (DEFIS) is used for learning the CFD results. The DEFIS learns the nanofluid velocity in the y-direction, as output, and the nodes coordinates (i.e., x, y, and z), as inputs. The intelligence of the DEFIS is assessed by adjusting the methd’s variables including input number, population number, and crossover. It was found that the DEFIS intelligence is related to the input number of 3, the crossover of 0.8, and the population number of 120. In addition, the nodes increment from 4833 to 774,468 was done by the DEFIS. The DEFIS predicted the velocity for the new dense mesh without using the CFD data. Finally, all CFD results were covered with the new predictions of the DEFIS.

## Introduction

Porous structures or metal foams have the potential of the heat transfer enhancement by the thermal conductivity and heat transfer area increment. A similar effect could be created by the proper suspension of the highly conductive nano solid particle in the working fluids^1^. These types of fluids known as nanofluids have shown the improved heat transfer properties for applications in energy transfer equipment^2–4^. Du et al.^5^ investigated the natural convection of ventilation cavity filling with the hybrid nanofluid (NF) of Cu–Al_2_O_3_/H_2_O and under the magnetic field. There are also a large number of studies that utilized a porous system in various conditions for improving heat transfer^6–9^. For example, an investigation indicated the improved heat transfer by reducing porosity^6^. The other study reported the enhancement of convective heat transfer by reducing porosity and increasing pore density^7^. It was also observed that the heat transfer increases by the flow’s inlet velocity due to rising the convective contribution in the heat transfer^8^.

Given that the particle size which constitutes the nanofluids is small, nanofluids can flow through a porous medium without blockage problem. A few studies have investigated forced convection heat transfer of different nanofluids in a porous medium^10–12^. Sureshkumar et al.^1^ recently showed heat transfer enhancement of MHD convection of a nanofluid in a cavity filled by porous media. Heat transfer enhancement of nanofluid flows in metal foams by nanoparticle concentration and Reynolds number has been observed by some studies^13,14^. The impact of the Darcy number, the dimension of porous medium, position, and Reynolds number on the improvement of alumina nanofluid’s heat transfer in a metallic porous structure was investigated by Siavashi et al.^15^. In the other study^16^, an improvement in heat transfer was reported for alumina nanofluid in a channel with a vertical arrangement and partial metal foam-filling. Nazari et al.^17^ analyzed the pressure loss and convection heat transfer behavior of alumina nanofluids in a porous metal foam tube. They achieved the highest pressure loss and the largest Nusselt number by 39% and 57%, respectively.

### Problem description

The CFD modeling could accurately predict the features of the fluid flow^18–20^. These predictions are helpful to prevent the costs coming from the try and error during the experiments. In addition, a number of the flow features cannot be achieved by the experimental measurements (e.g. velocity profile)^21^. In fact CFD computation is applicable here. But, the CFD techniques have their weaknesses, specifically in such a complicated phenomenon as turbulency, multi-phase flows, chemical reactions, etc. Newly some researchers have reported the ability of the algorithms based on artificial intelligence (AI) in the optimization of the complexity of the CFD approach^22–29^. These studies have considered just the adaptive network (AN) as a trainer of the CFD results in the combination with the fuzzy inference system (FIS). There does not exist investigations on the applicability of other types of trainers like differential evolution (DE) in combination with the FIS for simulating the reults obtained by CFD. Besides, adjustment of the tuning parameters of AI to implement for the case study, is not reported. Furthermore, the ability and efficiency of AI methods in mesh increments needs to be unlocked for application in physical processes.

Therefore, for filling the research gaps in this field and specifically for heat transfer improvements by nanofluids, this study tries to develop a new model of artificial intelligence in cooperation with the CFD to build a robust simulation methodology. Herein, the differential evolution (DE) algorithm is employed as the CFD data trainer in integration with the fuzzy inference system. Given that almost all studies through the literature have been dedicated to the nanofluid flow in conventional geometries and a few investigations exist about the nanofluids convective flow in a porous pipe, the velocity of Al_2_O_3_/water nanofluid flow in a porous pipe is considered as a simulation case study here. The intelligence of the developed AI algorithm of DEFIS is checked by tuning different parameters such as population number and crossover. Finally, the ability and efficiency of the intelligent DEFIS with the help of the CFD is shown. For the first time, the nodes increment and the fluid velocity prediction are made by the DEFIS without any requirements for performing CFD calculations.

## Methodology

### CFD technique

The analysis was done for 3D incompressible steady-state with turbulency in a pipe with porous media filling to rise the heat transfer rate. The porous media are saturated with a single-phase nanofluid. The basic hypothesis in the single-phase model is the mixture behaving as a single-phase system. The hypothesis is considered here for the numerical simulations. The NF in this model is treated as a normal fluid; however, its properties have been enhanced for the inclusion of NPs (Nanoparticles).

The main equations are expressed as:

Continuity, momentum, and energy equations^10,30,31^:

1 $$\nabla \cdot \left({\rho }_{nf}V\right)=0$$

2 $$\frac{1}{{\varepsilon }^{2}}\nabla \cdot \left({\rho }_{nf}\overrightarrow{V}\overrightarrow{V}\right)=-\nabla p+\frac{1}{\varepsilon }\nabla \left[{\mu }_{nf}^{e}\left(\nabla \overrightarrow{V}+{\left(\nabla \overrightarrow{V}\right)}^{T}\right)\right]-\frac{{\mu }_{nf}}{K}\overrightarrow{V}-\frac{{\varepsilon {C}_{d}\rho }_{nf}}{\sqrt{K}}\left|V\right|\overrightarrow{V}$$

3 $$\nabla \cdot \left({\rho }_{nf}V{C}_{p \cdot nf}T\right)=\nabla \cdot \left(\varepsilon {k}_{nf}\nabla T-{\varepsilon ({\rho C}_{p})}_{nf}\overline{VT}\right)$$

The parameters of porous media are taken from^32–35^.

### Al_2_O_3_/water properties

The required effective properties of the nanofluid considered in the simulations were given in Table 1. The effective density and specific heat of the NFs are a linear function of volume fraction of NPs in the fluid. Chon et al.^36^ correlation is employed for estimating the thermal conductivity of NF (Nanofluid). This correlation is used for Al_2_O_3_/water NF which takes into account the Brownian motion influence on thermal conductivity enhancement of NF.

**Table 1:** Al_2_O_3_/water properties.

Effective nanofluid properties	Correlation
Density^52^	${\rho }_{{nf}}=(1-{\alpha }){\rho }_{{f}}+{\alpha }{\rho }_{{np}}$
Heat capacity^52,53^	${{c}}_{{p},{nf}}=\frac{(1-{\alpha })(\rho {{c}}_{{p}}{)}_{{f}}+{\alpha }(\rho {{c}}_{{p}}{)}_{{np}}}{(1-{\alpha }){\rho }_{{f}}+{\alpha }{\rho }_{{np}}}$
Viscosity^52^	${\mu }_{{nf}}=(1+7.3{\alpha }+123{{\alpha }}^{2}){\mu }_{{f}}$
Thermal conductivity^53^	${{k}}_{{nf}}/{{k}}_{{bf}}=1+64.7{\left({\alpha }\right)}^{0.7460}{\left(\frac{{{d}}_{{bf}}}{{{d}}_{{np}}}\right)}^{0.3690}{\left(\frac{{{k}}_{{bf}}}{{{k}}_{{np}}}\right)}^{0.7476}{{Pr}}^{0.9955}{R}{{{e}}_{{np}}}^{1.2321}$ ${{Re}}_{{np}}=\frac{{\rho }_{{bf}}{{K}}_{{B}}{ T}}{3\pi {{\mu }_{{bf}}}^{2}\lambda }$

### Turbulence model

References of^37,38^ present $k-\varepsilon$ turbulence model for estimating the kinetic energy $(k)$, eddy viscosity, and its energy dissipation rate $(\varepsilon )$ as follows:

4 $$\nabla \cdot ({{\varvec{\rho}}}_{{\varvec{n}}{\varvec{f}}}{\varvec{k}}{\varvec{V}})=\nabla \cdot \left[(\frac{{{\varvec{\mu}}}_{{\varvec{t}}}}{{{\varvec{\sigma}}}_{{\varvec{k}}}})\nabla ({\varvec{k}}) \right]+{{\varvec{G}}}_{{\varvec{k}}}-{{\varvec{\rho}}}_{{\varvec{n}}{\varvec{f}}}{\varvec{\varepsilon}}$$

5 $$\nabla \cdot ({{\varvec{\rho}}}_{{\varvec{n}}{\varvec{f}}}{\varvec{\varepsilon}}{\varvec{V}})=\nabla \cdot \left[\frac{{{\varvec{\mu}}}_{{\varvec{t}}}}{{{\varvec{\sigma}}}_{{\varvec{\varepsilon}}}}\nabla{\varvec{\varepsilon}} \right]+\frac{{\varvec{\varepsilon}}}{{\varvec{k}}}({{\varvec{C}}}_{1{\varvec{\varepsilon}}}{{\varvec{G}}}_{{\varvec{k}}}-{{\varvec{C}}}_{2{\varvec{\varepsilon}}}{{\varvec{\rho}}}_{{\varvec{n}}{\varvec{f}}}{\varvec{\varepsilon}})$$

$${{\varvec{G}}}_{{\varvec{k}}}={{\varvec{\mu}}}_{{\varvec{t}}}(\nabla {\varvec{V}}+(\nabla {\varvec{V}}{)}^{{\varvec{T}}}),\boldsymbol{ }{{\varvec{\mu}}}_{{\varvec{t}}}={{\varvec{\rho}}}_{{\varvec{n}}{\varvec{f}}}{{\varvec{C}}}_{{\varvec{\mu}}}\frac{{{\varvec{k}}}^{2}}{{\varvec{\varepsilon}}}$$

$${C}_{\mu }=0.09, \; { \sigma }_{k}=1.00, \; { \sigma }_{\varepsilon } = 1.30, \; { C}_{1\varepsilon }=1.44,\; { C}_{2\varepsilon }=1.92$$

### Differential evolution algorithm (DE)

DE technique is an efficient optimization algorithm that is employed widely to solve combinatorial and continuous optimization problems^39^. This method begins with initializing a population. Trial offspring individuals are generated by choosing individuals as parents for crossover and mutation operators in this algorithm. A base individual is perturbed by a scaled difference vector mutation operation, in which the vector is composed of random individuals chosen from the population for generating a mutant individual. By comparing offspring individual and the parent in fitness value, the next generation’s new individual is created. With meeting a termination condition, the evolution process would be terminated. In the end, the best individual is the solution to the problem in the last generation. With initializing the population with the solution space’s individuals, evolutionary process is initiated by the DE algorithm. The individuals are designed as parents for crossover and mutation in each generation, and the trial offspring individuals are generated. A scaled differential vector perturbed the individual in the mutation phase, which possesses different individuals randomly chosen for producing the mutant individual. Then, there is a comparison of offspring individual with the parent by the use of fitness value. The best one will be selected as the next generation’s new individual. As the termination condition is met, the evolutionary process is ended, and the best individual of the last generation provides the problem’s solution^40–47^.

### Fuzzy inference system (FIS)

FIS is a well-known computing framework in different fields such a engineering and control systems. This approach relies on the concepts of fuzzy reasoning, set theory, and if–then logical aspect. Herein, to develop FIS for the NF, the common if–then logics are employed to build the FIS architecture for simulation of process and combination with other models The function assigned to the jth rule is expressed as:

7 $${w}_{j}=\alpha \left(Z\right) \beta \left(Y\right)\gamma (X)$$

where ${w}_{j}$ is the outcoming signal. Also, $\alpha , \beta , \gamma$ indicate the signals from membership functions (MFs) employed on inputs which in this case are Z-direction (Z), Y-direction (Y) and X-direction (X), to the designed node. More details of FIS are reported in^23,48–51,54^.

## Results and discussions

The potential of the nanofluids (NFs) for heat transfer augmentation has been confirmed already by experiments and computational studies. However, a few studies have considered the effect of metallic porous media on the flow characteristics as well as the heat transfer of the NFs. The porous media has shown a significant effect of the collapse of the hydraulic boundary layer of the fluids flow. So, the prediction of the velocity profile of the nanofluid flow in porous media can be important. The prediction of the velocity profile of the Al_2_O_3_/water nanofluid in aluminum porous pipe is considered for this paper. The CFD model using the finite volume method (FVM) is usually employed for such a prediction to obtain the parameters of interest. The CFD modeling can be more simplified by using AI. Herein, the differential evolution-based fuzzy inference system (DEFIS) learns the CFD results to find the pattern of the nanofluid velocity in the porous pipe. Havin determined the intelligence by the DEFIS model, further predictions of the NF velocity can be done for any boundary conditions and more nodes in the domain without the needs for CFD modeling.

**Figure 1:**
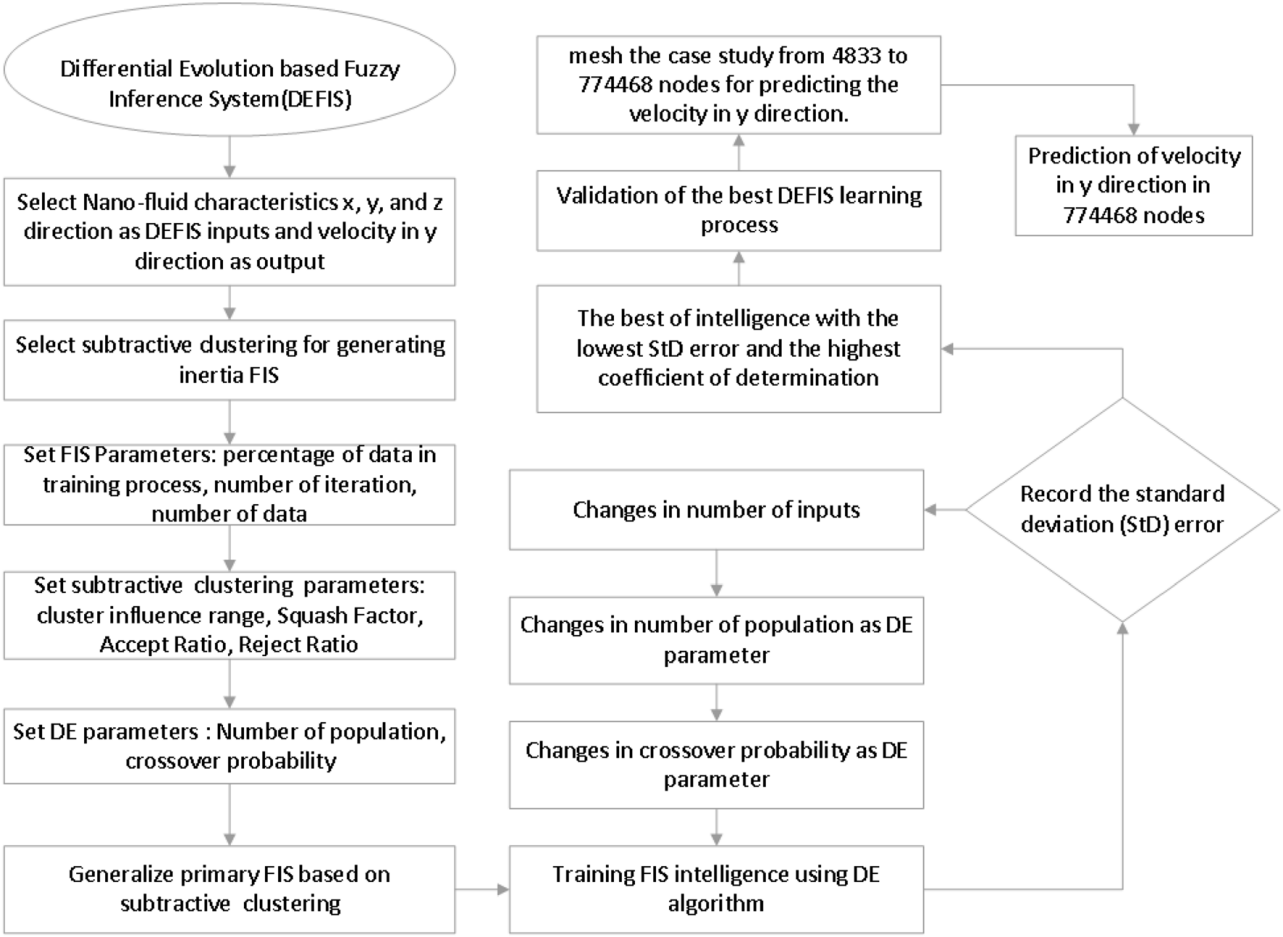
Flowchart of DEFIS method employed in this work.

The general processes of the DEFIS mechanism including the setup, the tuning analysis, validation, prediction, re-meshing, etc. are explained as a flowchart steply in Fig. 1. First of all, the position of the nodes (i.e. x, y, and z) are selected as the inputs, while the nanofluid velocity in the y-direction is the output. The data clustering is in kind of subtractive clustering and it is used for the inertia FIS. 70% of the total data number (i.e. 4833) is trained during 150 iterations. The algorithm of DE is adjusted by the evaluation of the proper population number and the crossover probability for different input numbers^54^. This adjustment could be led to the most precise of the DEFIS prediction calling the best intelligence. For this purpose, Table 2 summarizes the whole of DEFIS parameters that have been defined in this study. After learning the CFD data, the standard error (STD), and the coefficient of determination are calculated for different input numbers, population numbers, and probabilities. The DEFIS validation test is made by a comparison with the CFD results. Once the results have been validated, the node increment is done by the DEFIS without any CFD tool.

**Table 2:** Information of combining the FIS intelligence engine with DE algorithm.

Number of input in the best intelligence	3
Number of population in the best intelligence as a DE algorithm parameter	120
Crossover in the best intelligence as a DE algorithm parameter	0.8
Evaluation of number of inputs as a FIS parameter	2, 3
Evaluation of number of population as a DE algorithm parameter	60, 80, 100, 120
Evaluation of crossover as a DE algorithm parameter	0.1, 0.2, 0.3, 0.4, 0.5, 0.6, 0.7, 0.8
P (%) percentage of total data in training processes	70%
Number of data in learning process	4833
Number of predicted data	774,468
Number of iteration	150
Type of data clustering	Subtractive clustering
Type of membership function	guassmf
Cluster influence range (CIR)	0.2
Number of each input membership functions	135
Number of rules	135
Number of output membership functions	135

**Figure 2:**
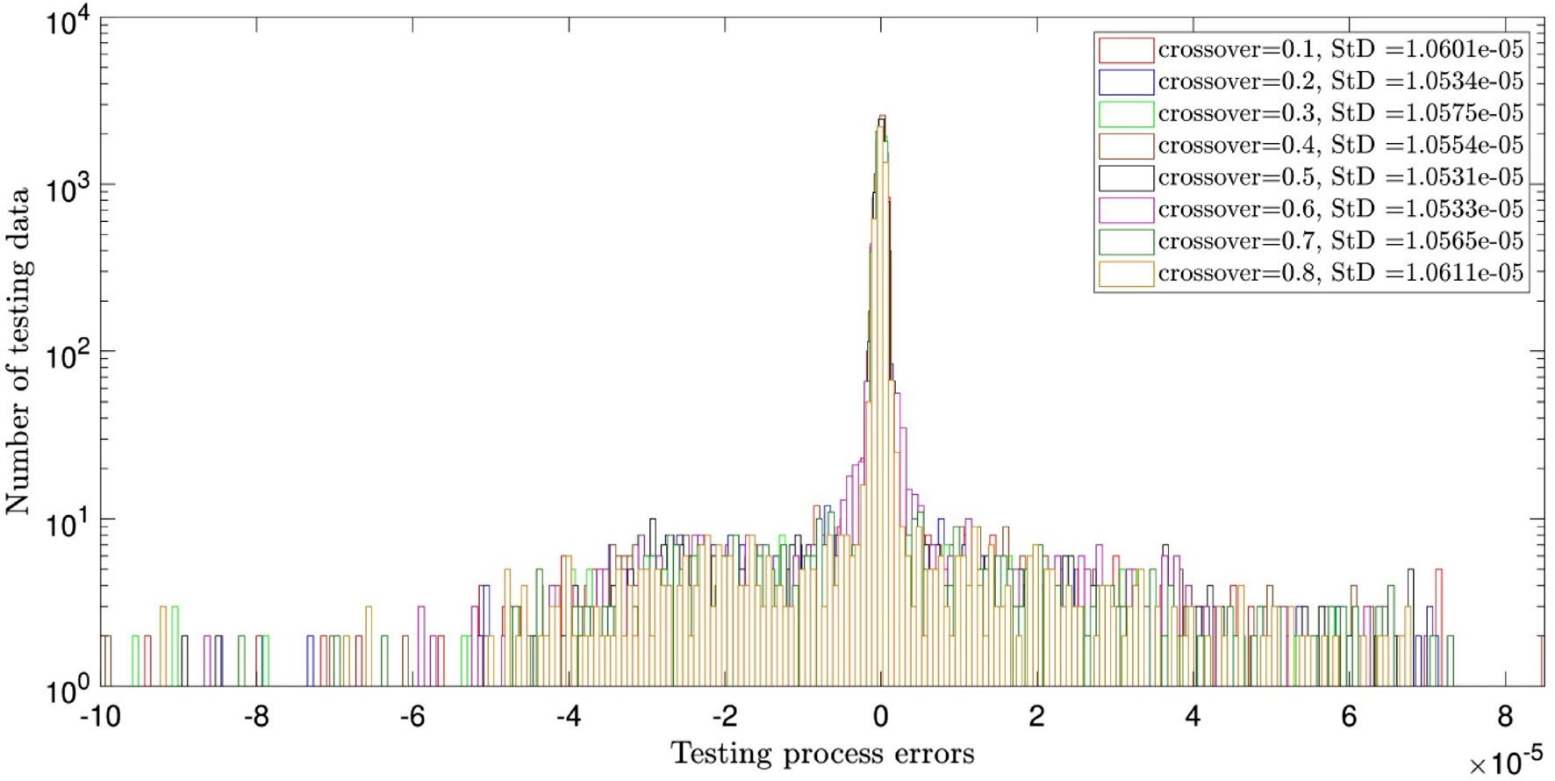
Standard deviation errors for different DEFIS learning processes considering different crossover with two inputs.

**Figure 3:**
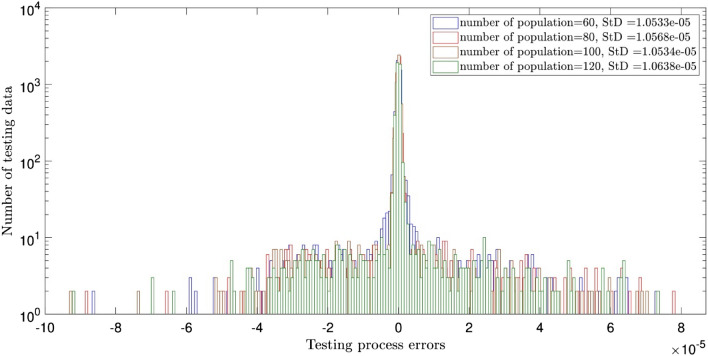
Standard deviation errors for different DEFIS learning processes considering different population with two inputs.

**Figure 4:**
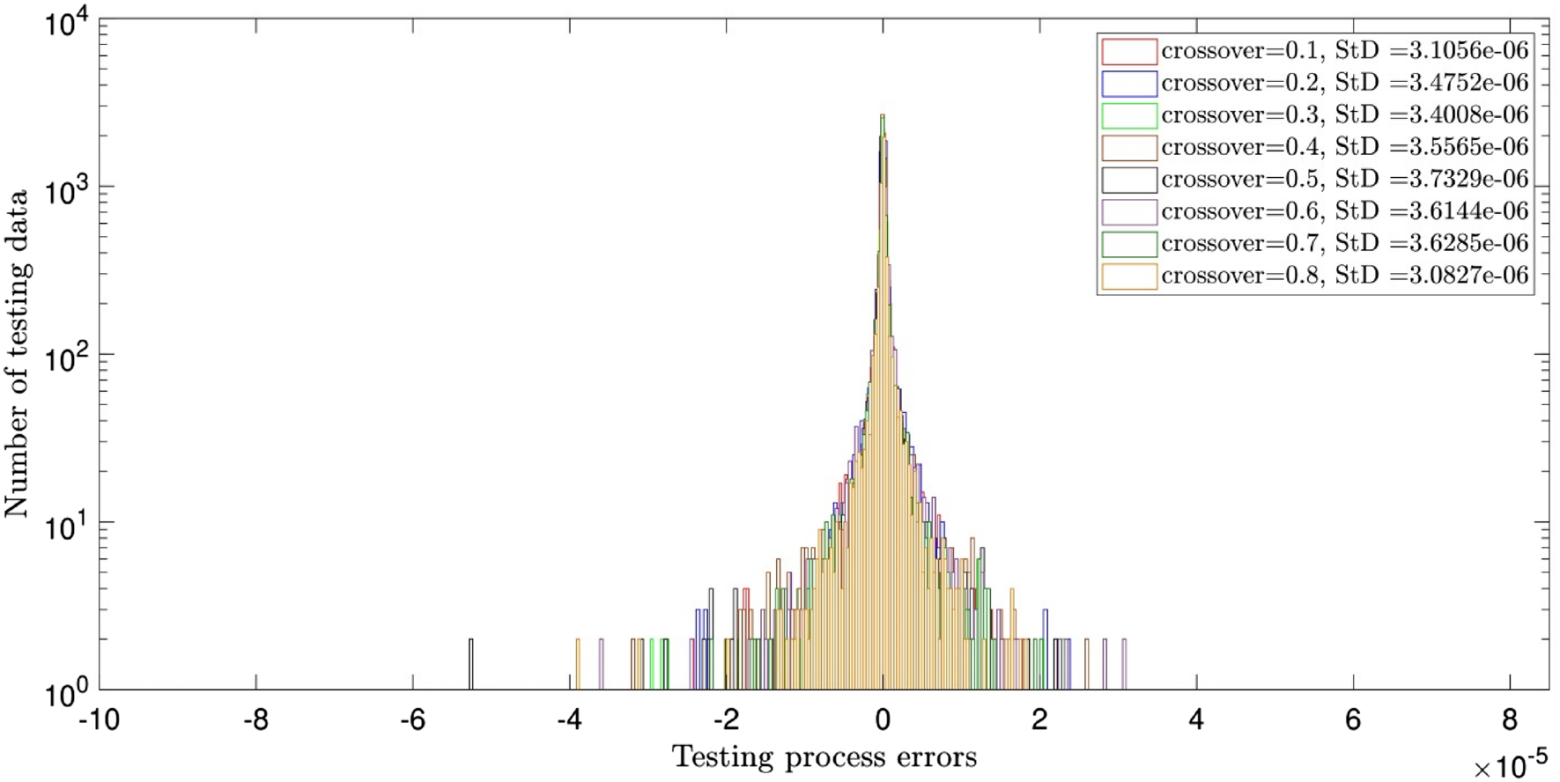
StD errors of learning processes considering different crossover with three inputs.

**Figure 5:**
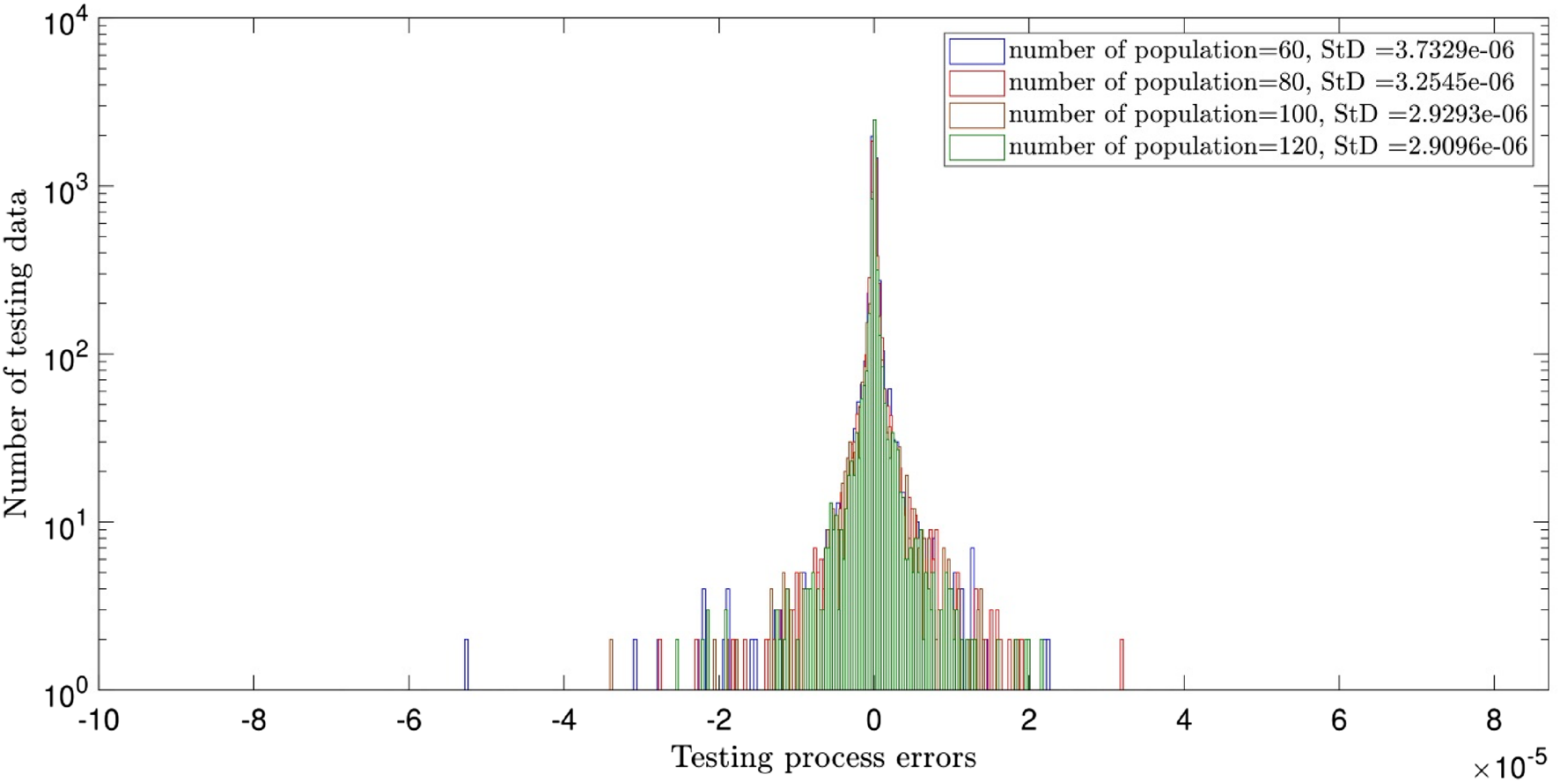
Standard deviation errors for different DEFIS learning processes considering different population with three inputs.

**Figure 6:**
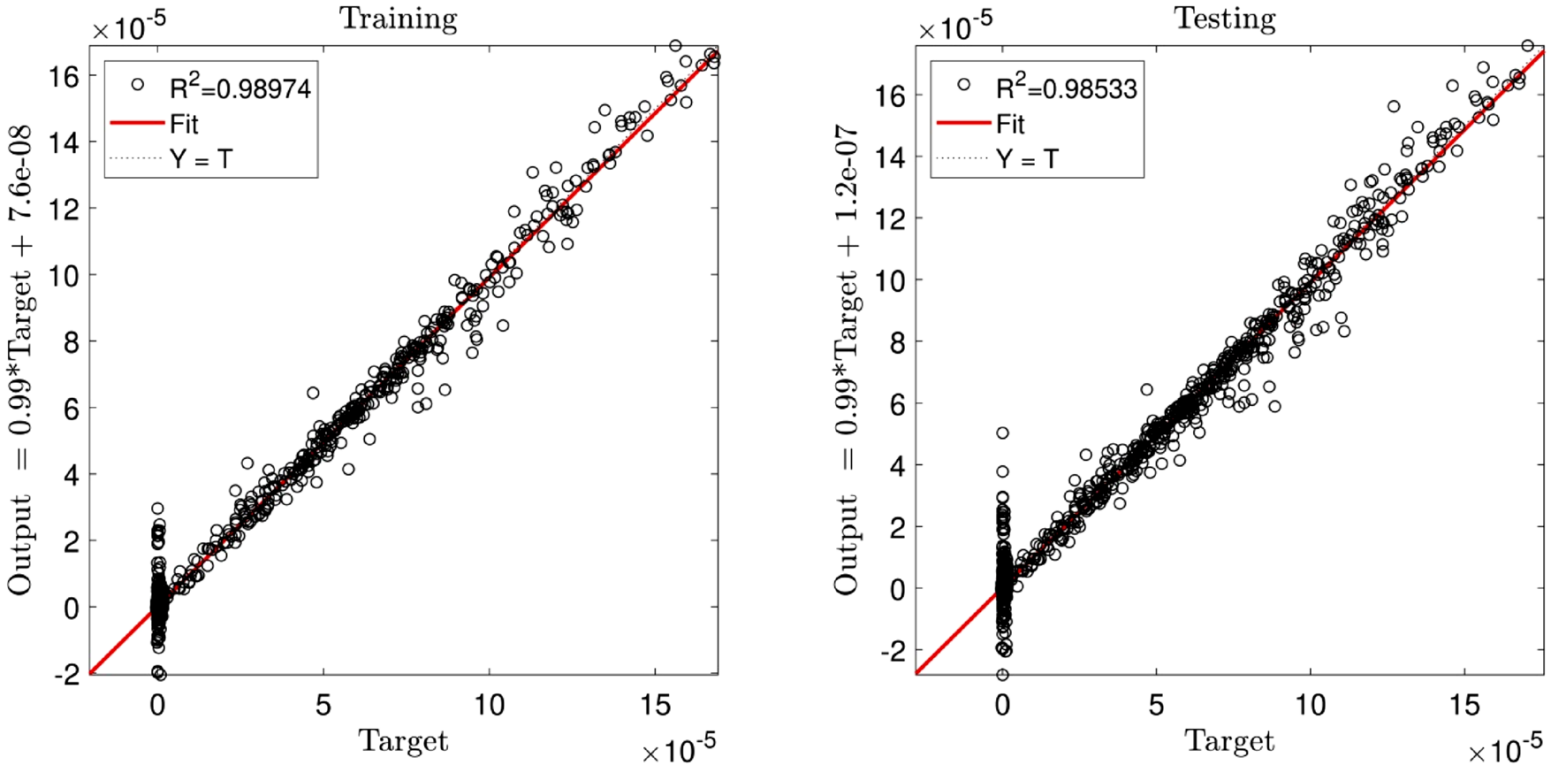
Coefficient of determination in the best DEFIS intelligence.

Figure 2 illustrates the errors distribution and StD errors for two inputs and the crossovers of 0.1, 0.2, 0.3, 0.4, 0.5, 0.6, 0.7, and 0.8. It is shown that the error distribution is so scattered between − 10 × 10^–5^ and 8 × 10^–5^. The StD is around 1.05 × 10^–5^. Similar analysis to Fig. 2, but for different population numbers of 60, 80, 100, and 120 is described in Fig. 3. The same error distribution and StD errors as Fig. 2 are seen in Fig. 3. Increasing the number of inputs to 3, almost all errors are distributed around zero (between ± 2 × 10^–5^) and the StD errors decline to around 3 × 10^–6^, as shown in Figs. 4 and 5. Totally, it is shown that the intelligence of DEFIS is more influenced by the number of inputs than the crossover and the population number. Changing in the crossover and the population number, a little change is seen in the accuracy of the DEFIS. In this case, the lowest StD is related to the crossover of 0.8 and the population number of 120. According to Fig. 6, the coefficient of determination is 0.98 for 3 inputs, the crossover of 0.8, and the population number of 120 (the optimal composition of input number, crossover, and population number for the best intelligence of DEFIS). It could be concluded that the tuning analysis is required for finding the effective parameters on the best intelligence of the DEFIS. In this study, the number of data learned by the DEFIS is equal to 4833. 70% of the data are trained, while the whole data are tested^55^.

**Figure 7:**
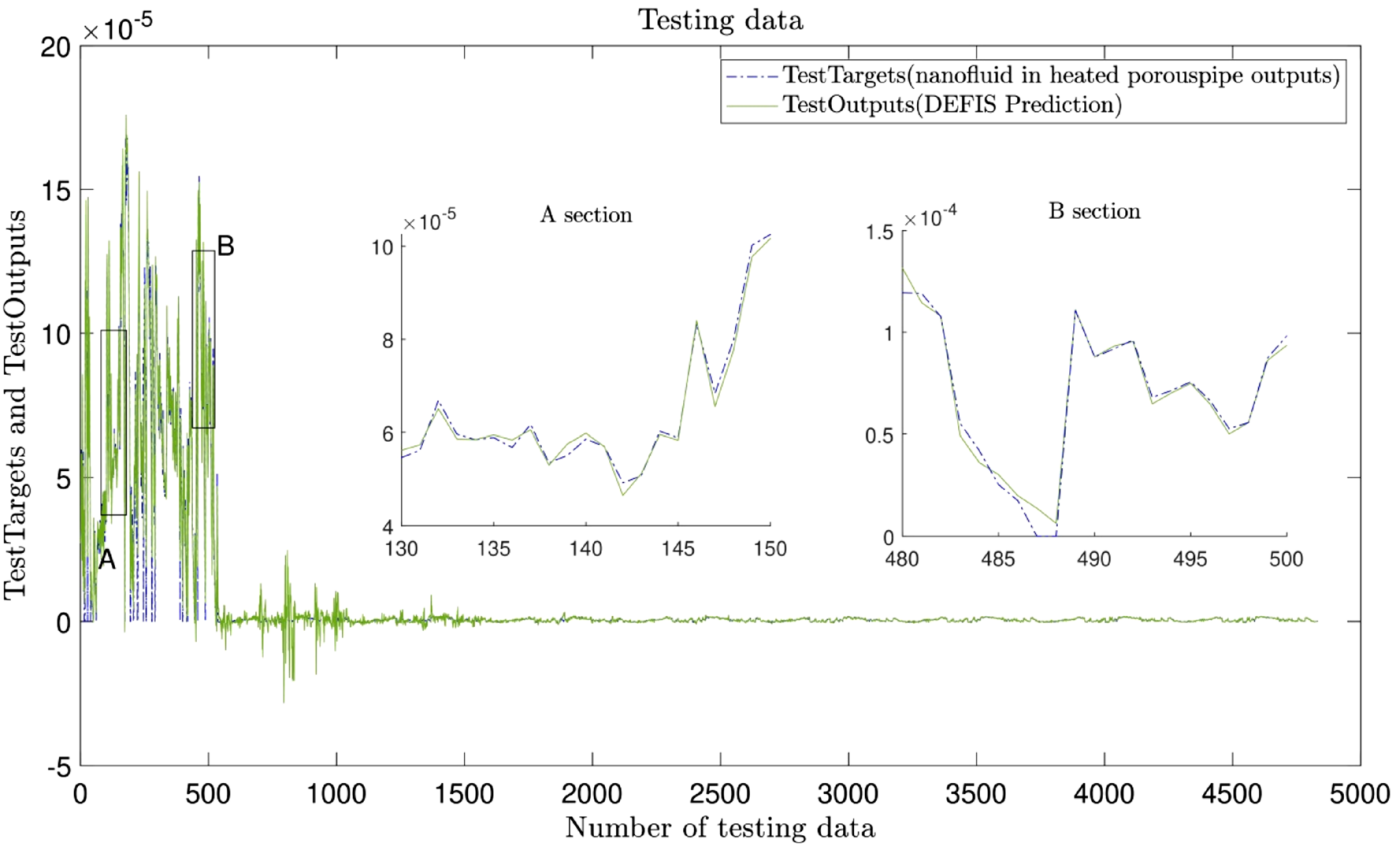
Validation of learning process.

**Figure 8:**
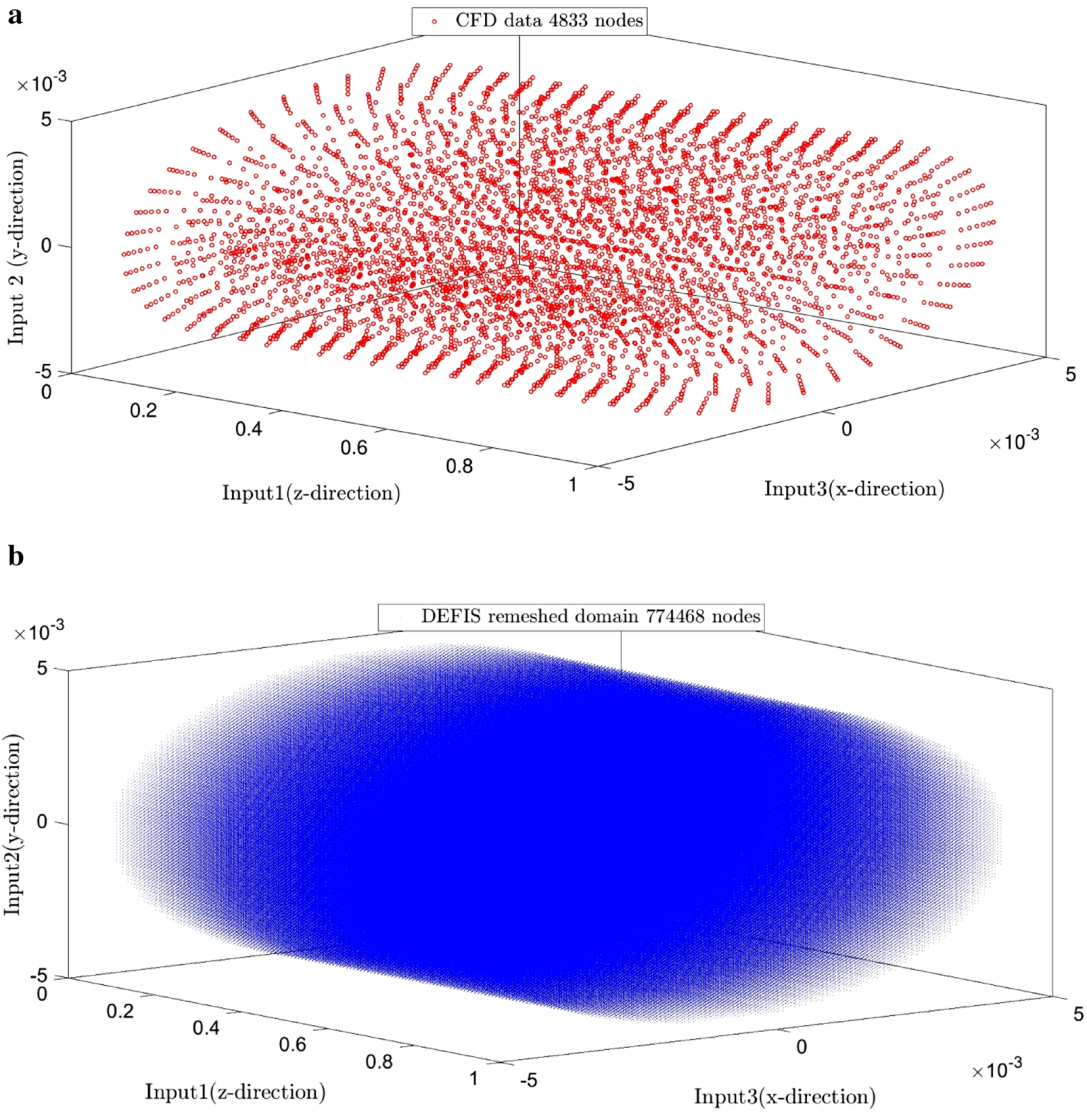
(**a**) CFD domain nodes. (**b**) Remeshed DEFIS domain nodes.

**Figure 9:**
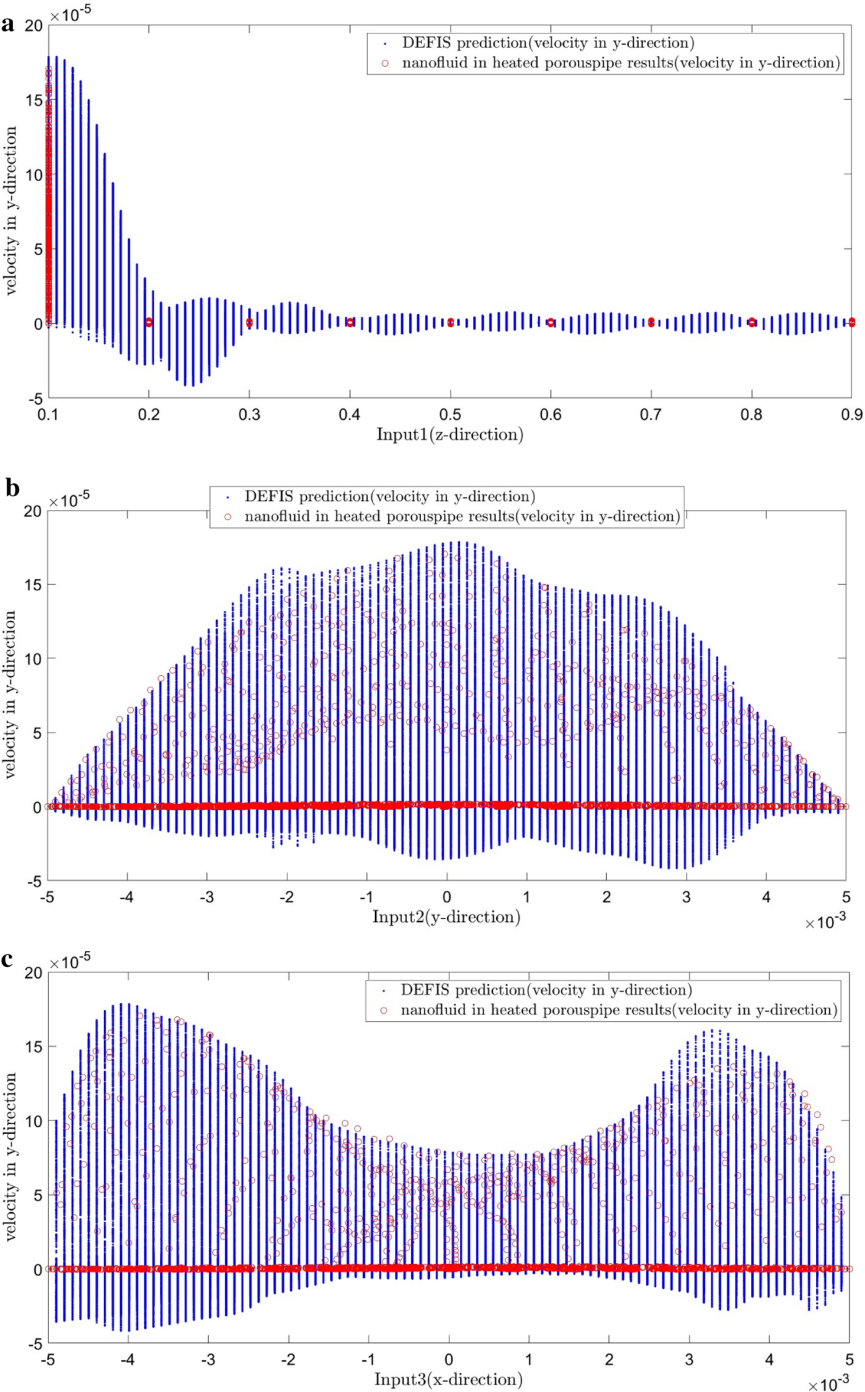
(**a**) DEFIS prediction (velocity in y direction) base first input (z-direction) in the remeshed 3D-domain from 4833 nodes to 774,468 nodes. (**b**) DEFIS prediction (velocity in y direction) base second input (y-direction) in the remeshed 3D-domain from 4833 nodes to 774,468 nodes. (**c**) DEFIS prediction (velocity in y direction) base third input (x-direction) in the remeshed 3D-domain from 4833 nodes to 774,468 nodes.

Figure 7 shows the comparison between the CFD results and the DEFIS ones as a validation. The values of the velocity of all nodes predicted by the CFD are the same as those predicted by the DEFIS. Negative velocities are also resulted by the DEFIS. These results are known as noises in the artificial algorithm predictions. Maybe it can be suggested to remove such noises by putting limitations as research investigation for future studies. The role of the DEFIS can be shown in Fig. 8 where the number of nodes increases remarkably from 4833 to 774,468. Creation such massive number of nodes and also CFD modeling for such dense mesh requires the consumption of a lot of time, computational expenses, and efforts. Machine learning (ML) of the DEFIS captures the overall pattern of the CFD results. Therefore, we become needless to solve a large number of complex governing equations. Figure 9 shows that the DEFIS could not only cover all results related to the CFD nodes, but it has also predicted the velocity for a lot of additional nodes. All these predictions have been done just by using DEFIS intelligence and without solving further CFD calculations. So, this optimizes the CFD approach and makes the computational predictions faster.

## Conclusions

The numerical predictions of the nanofluid flow characteristics in porous pipe haven't been fully investigated. The CFD tool can be time-consuming and needs expensive computational infrastructures especially in 3 dimensional cases, turbulent flows, and dense mesh models. Artificial intelligence algorithms have shown potential in data capturing. So, it was promising that the artificial intelligence algorithms could be helpful in facilitating the CFD approach. This study was aimed to show the ability and performance of the newly developed artificial intelligence of the DEFIS algorithm in the prediction of CFD simulations data. The CFD predicted the velocity of Al_2_O_3_/water nanofluid flow in a porous pipe. The DEFIS learned the CFD results for the prediction of the velocity. A sensitivity study was made to find the values of input number, population number, and crossover for the highest possible intelligence of DEFIS model. The results released that DEFIS intelligence is related to the input number of 3, the crossover of 0.8, and the population number of 120. At this condition, the coefficient of determination and the StD error was around 0.98 and 3 × 10^–6^ respectively. The DEFIS ability for mesh increment was also investigated. The nodes' increment from 4833 to 774,468 was done by the DEFIS. The DEFIS predicted the velocity for the new dense mesh without using the CFD. The nanofluid velocity predicted by the DEFIS covered all CFD results. This study showed the application of the DEFIS algorithm in cooperation with the CFD.

The results of this investigation could be extended to the Al_2_O_3_/water nanofluid flow in all straight pipe or tubes filling with porous media. In addition, the idea of this study including predictions of the CFD data and meshing processes by artificial intelligence algorithms could be used for other CFD cases.
